# Efficacy and safety of induction benmelstobart plus anlotinib and chemotherapy followed by concurrent chemoradiotherapy and benmelstobart consolidation versus concurrent chemoradiotherapy followed by benmelstobart consolidation in patients with unresectable stage III NSCLC: a randomized, controlled, multicenter phase II trial protocol (BEAM-LUNG)

**DOI:** 10.3389/fimmu.2026.1915557

**Published:** 2026-09-25

**Authors:** Zewen Song, Shuyun Ma, Xi Zhang, Xuewen Liu

**Affiliations:** Department of Oncology, The Third Xiangya Hospital of Central South University, Changsha, China

**Keywords:** anlotinib, benmelstobart, chemoradiotherapy, immunotherapy, induction therapy, non-small-cell lung cancer (NSCLC)

## Abstract

**Background:**

The PACIFIC and GEMSTONE-301 trials established consolidation immunotherapy post-chemoradiotherapy for unresectable stage III NSCLC, but 22–30% of patients fail initial chemoradiotherapy, precluding consolidation. PACIFIC-2 and CheckMate 73L failures highlight the need for better integration. Induction chemoimmunotherapy before radiotherapy may improve downstaging and immune activation. This trial evaluates whether induction benmelstobart (PD-L1 inhibitor) plus anlotinib (TKI) and chemotherapy, followed by concurrent chemoradiotherapy and benmelstobart consolidation, improves progression-free survival over standard concurrent chemoradiotherapy plus consolidation.

**Methods and analysis:**

This prospective, randomized, controlled, multicenter phase II trial will enroll 152 patients with histologically or cytologically confirmed unresectable stage III NSCLC (per UICC/AJCC 9th edition), without sensitizing driver mutations, and ECOG performance status 0–1. Randomization is centralized and stratified by PD-L1 tumor proportion score (<1% vs ≥1%), disease stage (IIIA vs IIIB/IIIC), histology, and participating centre. Patients will be randomized 1:1 to either the experimental arm (Arm A): two cycles of induction benmelstobart (1200 mg, day 1, every 3 weeks) plus anlotinib (8 mg, days 1–14) and platinum-based doublet chemotherapy, followed by concurrent chemoradiotherapy (with benmelstobart withheld during radiotherapy) and benmelstobart consolidation for up to one year; or the control arm (Arm B): concurrent chemoradiotherapy followed by benmelstobart consolidation for up to one year. The primary endpoint is progression-free survival (PFS) measured from randomization. Radiotherapy consists of involved-field IMRT/VMAT to a standard prescription of 60 Gy in 30 fractions, or a simultaneous integrated boost delivering 60–66 Gy to the planning gross tumor volume and 54 Gy to the planning target volume in 30 fractions; in the experimental arm, target volumes are based on post-induction residual disease with inclusion of all initially involved nodal stations. Consolidation benmelstobart will continue for up to one year or until disease progression/unacceptable toxicity. The sample size provides 80% power (two-sided α = 0.05) to detect a PFS hazard ratio of 0.55.

**Clinical trial registration:**

## Introduction

The treatment paradigm for unresectable stage III non-small-cell lung cancer (NSCLC) was revolutionized by the PACIFIC trial, which established concurrent chemoradiotherapy (cCRT) followed by consolidation durvalumab as the standard of care, demonstrating unprecedented survival benefits (1, 2). The subsequent GEMSTONE-301 trial further validated this approach in the Chinese population (3). However, despite these advances, several critical challenges remain.

First, a substantial proportion of patients—approximately 22% to 30%—are unable to receive consolidation immunotherapy due to disease progression or intolerable toxicity during the initial cCRT phase (4). This represents a fundamental limitation of the sequential PACIFIC regimen, leaving a significant patient population without access to the survival benefits of immunotherapy. Second, patients with PD-L1 expression <1% derive limited benefit from consolidation immunotherapy (5). Third, the high heterogeneity of stage III NSCLC means that even among those who receive consolidation therapy, survival outcomes remain variable (2).

To address these limitations, shifting immunotherapy earlier in the treatment sequence has been actively explored. The central hypothesis is that early immune activation could prime the tumor microenvironment, enhance systemic anti-tumor immunity, and potentially improve tumor control. However, the recent failures of the PACIFIC-2 (6) and CheckMate 73L (7) trials—which investigated the concurrent administration of immunotherapy with cCRT—underscore the complexity of this integration. These studies did not achieve superior outcomes compared to the standard PACIFIC sequence, with increased toxicity profiles being a major concern. These results refocus attention on a central scientific and clinical question: what is the optimal spatiotemporal integration of immunotherapy with definitive radiotherapy to maximize synergy while minimizing antagonism and toxicity?

Induction chemoimmunotherapy followed by definitive radiotherapy (with or without concurrent chemotherapy) has emerged as a promising alternative strategy to potentially circumvent the limitations of the PACIFIC model. Encouraging preliminary data from phase II studies and retrospective analyses suggest that this sequence may lead to improved tumor downstaging, higher response rates, and potentially better survival outcomes compared to cCRT followed by consolidation (8). Notably, a phase II study by Ross et al. demonstrated that atezolizumab before and after chemoradiation achieved a 24-month overall survival rate of 73.7%, comparing favorably to the 66.1% reported in the PACIFIC trial (9). Similarly, our multicenter retrospective data showed that the neoadjuvant immunotherapy plus chemotherapy group had significantly longer median PFS compared to the immunotherapy consolidation group (25.0 months vs. 16.3 months; HR = 0.57, 95% CI: 0.43–0.74; p < 0.001), with median OS not reached in the neoadjuvant group versus 41.1 months in the consolidation group (HR = 0.54, 95% CI: 0.37–0.78; p = 0.001) (10).

The R-ALPS trial has provided compelling evidence for the efficacy of benmelstobart-based consolidation therapy in this setting (11). This randomized, double-blind, placebo-controlled phase III study demonstrated that benmelstobart combined with anlotinib achieved a median PFS of 15.2 months, significantly superior to the 4.2 months observed with placebo (HR 0.49), while benmelstobart monotherapy achieved a median PFS of 9.7 months (HR 0.53). These results support benmelstobart with or without anlotinib as an effective option for consolidation therapy after chemoradiotherapy in unresectable stage III NSCLC. As the R-ALPS results are currently available in abstract form, the full peer-reviewed publication is awaited; benmelstobart has already received a new indication approval from China’s National Medical Products Administration (NMPA) for unresectable stage III NSCLC after platinum-based concurrent or sequential chemoradiotherapy; benmelstobart is also approved in China in combination with anlotinib and chemotherapy for extensive-stage small-cell lung cancer (12) and has shown efficacy versus pembrolizumab in PD-L1-positive advanced NSCLC (CAMPASS) (13).

Building upon these foundations, we hypothesize that moving immunotherapy and targeted therapy even earlier—into the induction phase—could further improve outcomes. The addition of anlotinib to induction therapy is mechanistically rational. Anlotinib is a multi-target tyrosine kinase inhibitor of VEGFR1–3, FGFR1–4, PDGFR-α/β, c-Kit, and Ret; beyond its direct anti-angiogenic effect, it downregulates PD-L1 expression on tumor-associated vascular endothelial cells, thereby relieving vascular immune suppression and remodeling the tumor immune microenvironment with enhanced T-cell infiltration (14, 15). In NSCLC patients treated with neoadjuvant anlotinib plus PD-1 blockade, single-cell transcriptomics demonstrated that VEGF-signaling-mediated reprogramming of the tumor vasculature underlies the response to this combination (14, 15), and autophagy modulation further potentiates the synergy between anlotinib and PD-1 blockade in preclinical models (16). These vascular- and immune-remodeling effects are expected to be most effective when delivered before chemoradiotherapy, while the tumor vasculature is intact and before radiation-induced lymphodepletion, providing a biological rationale for induction-phase rather than concurrent or consolidation-phase anlotinib (14, 15).

Therefore, we propose a comprehensive strategy comprising induction benmelstobart plus anlotinib and platinum-based doublet chemotherapy, followed by definitive concurrent chemoradiotherapy (with benmelstobart withheld during radiotherapy to mitigate potential toxicity), and subsequent benmelstobart consolidation therapy. This approach aims to synergistically achieve deep tumor response through multi-modality induction, enhance the abscopal effect through immune priming, and minimize treatment-related toxicities by temporally separating immunotherapy from radiotherapy.

The BEAM-LUNG trial is a prospective, randomized, controlled phase II study designed to test the efficacy and safety of this integrated treatment strategy compared to the standard cCRT followed by benmelstobart consolidation regimen.

## Methods and analysis

### Study design

The BEAM-LUNG trial is a prospective, randomized, controlled, multicenter, open-label phase II study designed to evaluate the efficacy and safety of an integrated treatment strategy for patients with unresectable stage III NSCLC (**Figure 1**). This strategy consists of induction benmelstobart plus anlotinib and chemotherapy, followed by definitive concurrent chemoradiotherapy (with benmelstobart withheld during radiotherapy), and culminating in benmelstobart consolidation therapy. A total of 152 eligible patients will be enrolled across multiple centers in China (the final number of participating centers and expected recruitment per center are provided in the **Supplementary Material**) and randomized 1:1 to either the experimental arm or the control arm. A SPIRIT-compliant schedule of enrolment, interventions, and assessments is provided in **Supplementary Figure S1**.

**Figure 1:**
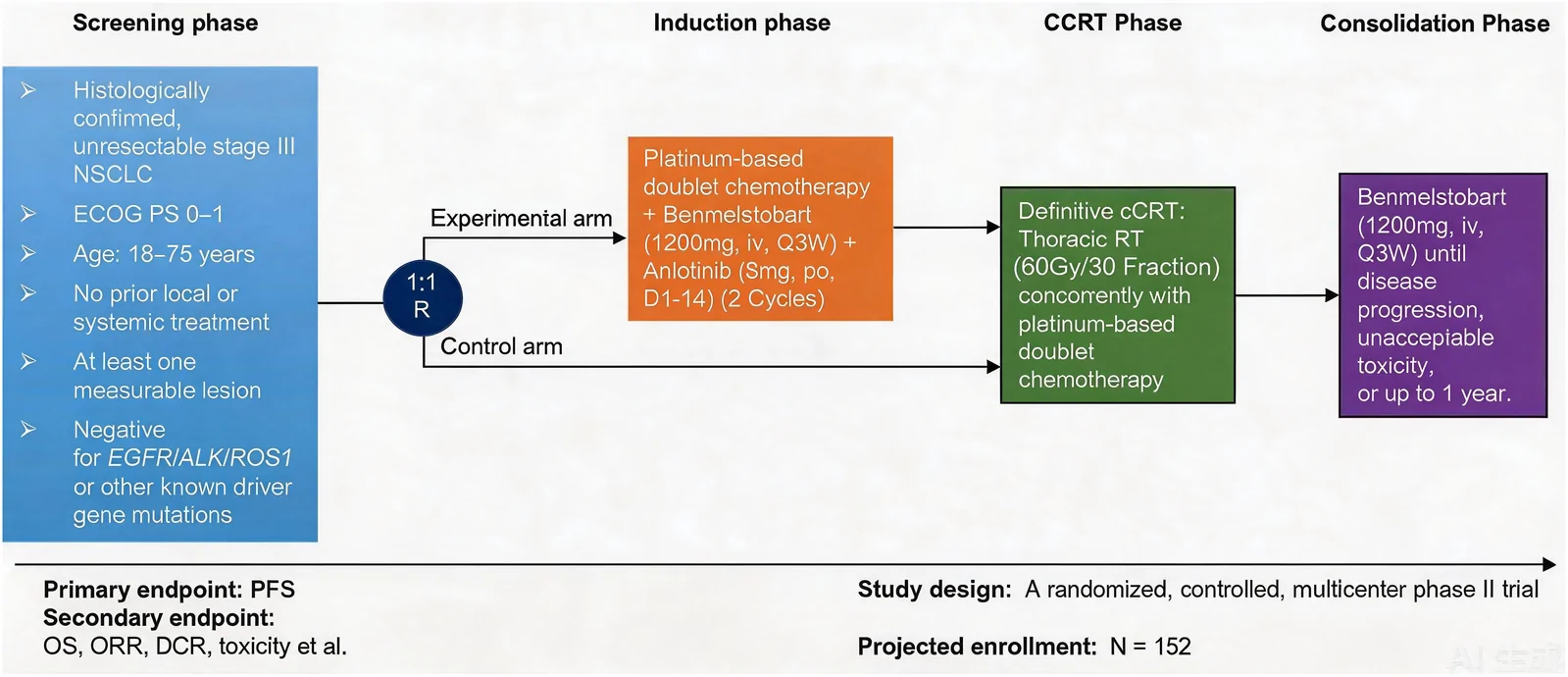
Study design and treatment schema. Eligible patients with unresectable stage III NSCLC are randomized 1:1 to receive either (Experimental arm) two cycles of induction benmelstobart plus anlotinib and platinum-based chemotherapy, followed by definitive cCRT (with benmelstobart withheld) and benmelstobart consolidation; or (Control arm) definitive cCRT followed by benmelstobart consolidation. Radiotherapy is delivered as involved-field IMRT/VMAT to 60 Gy in 30 fractions (or a simultaneous integrated boost of 60–66 Gy to the PGTV and 54 Gy to the PTV in 30 fractions). The primary endpoint is PFS; secondary endpoints include OS, ORR, DCR, and toxicity. Total projected enrollment: N= 152.

Following confirmation of unresectable status by a multidisciplinary team, patients in the experimental arm (Arm A) will receive two cycles of induction therapy (benmelstobart + anlotinib + platinum-based doublet chemotherapy), followed by concurrent chemoradiotherapy (cycles 3–4, with benmelstobart withheld), and then benmelstobart consolidation for up to one year. Patients in the control arm (Arm B) will receive concurrent chemoradiotherapy followed by benmelstobart consolidation for up to one year.

#### Randomization and stratification

Eligible patients are randomized 1:1 through a centralized interactive web response system with permuted-block randomization prepared by an independent statistician. Allocation is stratified by PD-L1 tumor proportion score (TPS; <1% vs ≥1%), disease stage (IIIA vs IIIB–IIIC, 9th edition), histology (squamous vs non-squamous), and participating centre.

#### Definition of unresectability

Unresectable disease is defined prospectively as stage III NSCLC for which an R0 resection cannot be safely achieved, as determined by a multidisciplinary tumor board that must include experienced thoracic surgeons, medical radiation and medical oncologists, pathologists, diagnostic radiologists, nuclear medicine physicians and pulmonologists who experienced in stage III disease. The board documents the specific reason for unresectability (multistation or bulky N2 disease, N3 disease, T4 invasion, or comorbidity-related contraindications). Patients with potentially resectable, single-station, non-bulky N2 disease are eligible only when the tumor board documents a consensus judgment of unresectability; the resectability category is recorded and used as a predefined subgroup variable.

#### Analysis populations

The intention-to-treat (ITT) population—all randomized patients analyzed according to the assigned arm—is the primary efficacy population. The per-protocol population comprises ITT patients who receive the assigned treatment without major protocol deviations, and the safety population comprises all patients who receive at least one dose of study treatment. Patients who never start chemoradiotherapy, undergo off-protocol surgery, or change systemic treatment are analyzed as randomized within the ITT analysis, with prespecified sensitivity analyses (see Statistical Analysis).

### Research objectives

#### Primary objective

To compare progression-free survival (PFS) between the two arms, defined as the time from the date of randomization to the first documented disease progression according to RECIST v1.1 or death from any cause. Radiological assessments are performed at identical scheduled intervals from randomization in both arms, and imaging for the primary endpoint will be adjudicated by blinded independent central review (BICR).

#### Secondary objectives

To compare overall survival (OS), objective response rate (ORR), disease control rate (DCR), duration of response (DoR), in-field local control rate, out-of-field recurrence rate, and the incidence and severity of adverse events between the two arms. Marginal and out-of-field recurrences are defined based on delivered dose distribution per ICRU Report 83 and RTOG trial conventions: In-field local control is defined at the patient level as recurrence within the 50% isodose surface (relative to the prescription dose) of the delivered radiotherapy plan; marginal recurrences (within the region receiving 20–50% of the prescription dose) and out-of-field recurrences (<20%) are reported separately, and patients with multiple simultaneous progression sites are counted once per endpoint. The duration and schedule of post-consolidation follow-up are prespecified in the protocol.

#### Exploratory objectives

- To assess health-related quality of life (HRQoL) using standardized questionnaires (EORTC QLQ-C30, QLQ-LC29, EQ-5D-5L. The principal HRQoL domain (QLQ-C30 global health status at 24 weeks after randomization), assessment time points, compliance reporting, handling of missing questionnaires, thresholds for clinically meaningful change, and the longitudinal analysis model are prespecified in the statistical analysis plan.).
- To evaluate correlations between treatment response and genomics, proteomics, single-cell sequencing, and gut microbiome profiles derived from tumor tissue, blood, and stool samples. Translational analyses are classified *a priori* as hypothesis-driven—ctDNA molecular response after induction and after chemoradiotherapy and its association with PFS—or exploratory (genomics, proteomics, single-cell sequencing, immune profiling, and gut microbiome); sample-collection time points, laboratory standardization, tissue-adequacy requirements, storage, batch-effect control, analytical populations, and multiplicity considerations are detailed in the translational research appendix, and exploratory analyses are considered hypothesis-generating.

### Participants

#### Main inclusion criteria

1. Voluntary signing of written informed consent.
2. Age 18-75 years, male or female.
3. ECOG performance status 0 or 1.
4. Life expectancy ≥3 months.
5. Histologically or cytologically confirmed stage III NSCLC (per UICC/AJCC 9th edition). Staging must include contrast-enhanced brain MRI, contrast-enhanced CT of the neck-thorax-abdomen, and a whole-body bone scan or FDG-PET/CT be completed within 28 days before randomization. Whole-body FDG-PET/CT is strongly preferred. Within 28 days before randomization, with pathological confirmation of radiologically suspicious mediastinal lymph nodes by EBUS, EUS, or mediastinoscopy whenever clinically feasible. The maximum intervals from staging to randomization, from randomization to the start of study treatment (≤7 days), from the end of induction to restaging (≤7 days), and from restaging to radiotherapy simulation and the start of chemoradiotherapy (≤3 weeks) are prespecified.
6. Confirmed unresectable disease by multidisciplinary tumor board discussion, including at least one experienced thoracic surgeon (see Study Design).
7. No sensitizing driver gene alterations, defined as EGFR-sensitizing mutations, ALK, ROS1, RET, NTRK, BRAF V600E, MET exon 14 skipping, or HER2 alterations. Driver testing before randomization is required for all non-squamous NSCLC; for squamous NSCLC, testing is required for never-smokers or patients with a smoking history of <15 pack-years, and is performed whenever tissue is available for other patients. PD-L1 expression (tumor proportion score) must be assessed before randomization by a validated immunohistochemical assay (e.g., 22C3 or SP263) on tumor tissue; local testing is acceptable. PD-L1 status is not an eligibility criterion but is a randomization stratification factor.
8. No prior systemic anti-tumor therapy or thoracic radiotherapy for NSCLC.
9. At least one measurable lesion per RECIST v1.1.
10. Provision of tumor tissue samples (archived or fresh) for biomarker analysis (10-25 FFPE sections or fresh frozen tissue).
11. Adequate organ function as defined by protocol-specified laboratory parameters.
12. Effective contraception for women of childbearing potential and men with partners of childbearing potential.

#### Key exclusion criteria

1. Presence of any small-cell carcinoma component or special histological types (e.g., salivary gland-type, SMARCA4-deficient).
2. Other malignancies within 5 years prior to enrollment (except cured non-melanoma skin cancer, superficial bladder cancer, cervical or breast carcinoma *in situ*).
3. Concurrent enrollment in another clinical study (except observational studies or follow-up phases).
4. Prior thoracic radiotherapy or local ablative therapy to tumor lesions.
5. Received non-specific immunomodulatory therapy within 2 weeks prior to first dose, or anti-tumor Chinese herbal medicine within 1 week.
6. Active autoimmune disease requiring systemic treatment within the past 2 years.
7. Immunodeficiency; HIV antibody positive; currently on long-term systemic corticosteroids or other immunosuppressants.
8. Active tuberculosis or active syphilis.
9. History of allogeneic organ or hematopoietic stem cell transplantation.
10. History of or current non-infectious pneumonitis/interstitial lung disease requiring systemic corticosteroids.
11. Severe infection within 4 weeks prior to first dose, or active infection requiring systemic anti-infective therapy within 2 weeks.
12. Active hepatitis B (HBsAg positive with HBV DNA >1000 copies/mL) or active hepatitis C (HCV antibody positive with HCV-RNA above lower limit of detection).
13. Major surgery or severe trauma within 30 days prior to first dose, or planned major surgery within 30 days after first dose.
14. Tumor invasion or compression of vital organs (e.g., aorta, heart and pericardium, superior vena cava, trachea, esophagus) or risk of esophagotracheal or esophagopleural fistula.
15. Uncontrolled comorbidities including decompensated cirrhosis, nephrotic syndrome, uncontrolled metabolic disorders, severe active peptic ulcer disease or gastritis.
16. History of myocarditis, cardiomyopathy, malignant arrhythmia; unstable angina, myocardial infarction, congestive heart failure, or vascular disease requiring hospitalization within 12 months prior to first dose.
17. History of esophagogastric varices, severe ulcer, non-healing wound, gastrointestinal perforation, abdominal fistula, gastrointestinal obstruction, intra-abdominal abscess, or acute gastrointestinal bleeding within 6 months prior to first dose; acute exacerbation of COPD within 1 month.
18. Any arterial thromboembolic event, grade ≥3 venous thromboembolic event, transient ischemic attack, cerebrovascular accident, hypertensive crisis, or hypertensive encephalopathy within 6 months prior to first dose; uncontrolled hypertension with systolic BP ≥160 mmHg or diastolic BP ≥100 mmHg.
19. History of bleeding diathesis or coagulation dysfunction; clinically significant hemoptysis within 1 month prior to first dose; imaging showing tumor encasement of major vessels or significant necrosis/cavitation with bleeding risk.
20. Received live vaccine within 30 days prior to first dose, or plans to receive live vaccine during study.
21. Known allergy to any component of study drugs; history of severe hypersensitivity to other monoclonal antibodies.
22. Known psychiatric disorders, substance abuse, alcoholism, or drug abuse history.
23. Pregnancy or breastfeeding.
24. Any disease, treatment, or laboratory abnormality that may confound study results or interfere with participation.
25. Non-malignant systemic disease or tumor-related conditions that may increase medical risk or affect survival assessment.

### Treatments

The treatment protocol comprises three distinct phases: induction therapy (Experimental arm only), concurrent chemoradiotherapy, and consolidation therapy.

#### Induction phase (experimental arm, cycles 1-2)

Patients in Arm A receive two cycles of induction therapy. Each 21-day cycle consists of: - Benmelstobart 1200 mg administered intravenously on day 1. - Anlotinib 8 mg orally on days 1-14. - Platinum-based doublet chemotherapy (the same regimen continued during the concurrent phase; see below). A minimum washout interval of 7 days (approximately two half-lives of anlotinib) is required between the last anlotinib dose and the start of radiotherapy, and radiotherapy begins only after post-induction imaging excludes new tumor cavitation, fistula, or vascular encasement.

##### Post-induction reassessment and conversion to resectability

All patients in Arm A undergo protocol-specified restaging within 7 days after the end of cycle 2 and are re-reviewed by the multidisciplinary tumor board. Per protocol, treatment continues with definitive chemoradiotherapy irrespective of the degree of response; conversion surgery is not permitted as protocol treatment. Patients who proceed to surgery off protocol remain in the ITT population and are analyzed as randomized; surgery is documented as a major protocol deviation, and post-operative management including whether to resume benmelstobart is left to the investigator’s discretion. Given that benmelstobart has no approved peri-operative indication, management should follow standard-of-care regimens, is at the discretion of the treating physician. In the event of a radiological complete response after induction, definitive chemoradiotherapy is still delivered, with the target volume reconstructed from co-registered pre-induction imaging so that all initially involved sites are covered. Throughout the manuscript, tumor shrinkage (radiological response), downstaging (reduction in AJCC category), and conversion to technical resectability are distinguished.

#### Concurrent chemoradiotherapy phase (cycles 3-4)

Both arms receive concurrent chemoradiotherapy. Radiotherapy is delivered concurrently with two additional cycles (cycles 3 and 4) of chemotherapy. Importantly, benmelstobart is withheld during radiotherapy in both arms. This decision is based primarily on efficacy: randomized trials have consistently failed to show a benefit of adding concurrent checkpoint inhibition to chemoradiotherapy (PACIFIC-2, CheckMate 73L, and ECOG-ACRIN EA5181) (6, 7, 17), while the safety argument (radiation pneumonitis and lymphopenia) provides additional support.

Chemotherapy Regimens. All chemotherapy is administered at full systemic doses. The platinum doublet selected for induction must be continued during concurrent chemoradiotherapy in Arm A; in both arms, chemotherapy is administered on a 21-day cycle for two cycles (cycles 3–4).

1. Non-adenocarcinoma (squamous and other NSCLC histologies): nab-paclitaxel 100 mg/m^2^ on days 1, 8, and 15, paclitaxel 135–175 mg/m^2^ on day 1, or liposomal paclitaxel 135–175 mg/m^2^ on day 1, plus either cisplatin 75 mg/m^2^ on day 1 or carboplatin AUC 5 on day 1. The three taxane formulations are considered therapeutically equivalent and are permitted interchangeably based on institutional availability and patient factors.
2. Adenocarcinoma: pemetrexed 500 mg/m^2^ on day 1, plus either cisplatin 75 mg/m^2^ on day 1 or carboplatin AUC = 5 on day 1. Pemetrexed is administered with mandatory folic acid and vitamin B12 supplementation and dexamethasone premedication as per standard institutional practice. Dose reductions (by one dose level) and treatment delays (up to 2 weeks) are permitted for hematologic and neurological toxicity according to protocol-specified modification rules, and cumulative toxicity is managed per CTCAE v5.0-based guidance in the full protocol. The chemotherapy regimen and platinum compound are recorded and included as covariates in the primary analysis model.

### Radiotherapy planning and delivery

Radiotherapy target volumes are defined according to ICRU Reports 62 and 83. Elective nodal irradiation is prohibited. In the experimental arm, all nodal stations meeting CT--based metastatic criteria, demonstrating FDG avidity, or pathologically proven involved prior to induction therapy are included within the clinical target volume (CTV). FDG--PET/CT--informed radiotherapy planning is prioritized when both economic and technical conditions permit, which is in line with the PET--Plan trial (18). The PGTV (GTV + 0.5 cm) denotes the planning boost volume used for simultaneous integrated boost prescription. Gross Tumor Volume (GTV): Primary tumor and involved positive lymph nodes. - Planning GTV (PGTV): GTV + 0.5 cm margin. - Clinical Target Volume (CTV): GTV expanded with histology-specific margins: 0.6 cm for squamous cell carcinoma, 0.8 cm for adenocarcinoma; 0.3 cm for positive lymph nodes with short axis <2 cm, 0.5 cm for nodes with short axis ≥2 cm. CTV is manually edited to respect anatomical barriers. - Planning Target Volume (PTV): CTV + 0.5 cm margin.

Organs at risk (lungs, heart, esophagus, spinal cord, proximal bronchial tree, and brachial plexus) are delineated according to the RTOG 1106 atlas, with the following mandatory constraints: lungs V20 ≤25%, V5 ≤50%, Dmean ≤14 Gy; spinal cord Dmax ≤45 Gy; esophagus Dmean ≤34 Gy; heart Dmean ≤26 Gy and V30 ≤45%; proximal bronchial tree Dmax ≤70 Gy; brachial plexus Dmax ≤66 Gy. The lung constraints (V20 ≤25%, stricter than the conventional V20 ≤35%) were prespecified because patients in the experimental arm have received prior anlotinib and platinum-based chemotherapy, both of which may increase the risk of radiation pneumonitis and pulmonary vascular injury. When target coverage and organ-at-risk constraints conflict, the mandatory organ-at-risk constraints take precedence: the prescribed dose is reduced and/or the target volume re-contoured to satisfy them; patients whose plans cannot meet the mandatory constraints after renormalization are not eligible to proceed with protocol radiotherapy.

The standard radiotherapy prescription is 60 Gy in 30 fractions of 2.0 Gy to the PTV. Alternatively, a simultaneous integrated boost may be used (PGTV 60–66 Gy in 30 fractions with PTV 54 Gy in 30 fractions); in this scheme, gross residual disease and all initially involved nodal stations receive the PGTV dose. The previously permitted single-PTV prescription range of 54–66 Gy has been discontinued to reduce inter-center heterogeneity, and a definitive dose below 60 Gy to gross disease is not permitted. 

All radiotherapy is delivered using IMRT or VMAT techniques with daily image guidance (cone-beam CT). Four-dimensional CT simulation is used for patients with significant respiratory motion, with internal target volume margins applied as appropriate; immobilization and motion management follow institutional standards, and adaptive replanning is permitted for marked tumor regression or anatomical change.

#### Radiotherapy quality assurance

For this multicenter trial, prospective quality assurance comprises: (1) institutional credentialing including IMRT/VMAT capability and dosimetry audit; (2) completion of a benchmark case before center activation; (3) prospective central review of contours and treatment plans for the first two patients at each center and on a sampling basis thereafter; and (4) predefined criteria for major and minor deviations in target coverage and organ-at-risk constraints, with deviation rates reported in the final publication.

#### Consolidation phase

Within 4 weeks after the completion of radiotherapy, patients who have not experienced disease progression or unacceptable toxicity begin consolidation therapy with benmelstobart monotherapy. Benmelstobart 1200 mg is administered intravenously every 3 weeks for up to one year, or until disease progression, unacceptable toxicity, or withdrawal of consent.

### Assessment

Tumor response will be assessed by contrast-enhanced CT (with brain MRI) per RECIST v1.1. Radiological assessments are performed at identical scheduled intervals from randomization in both arms: at screening (within 28 days before randomization), at week 7 (± 1 week) after randomization (in Arm A this assessment serves as post-induction restaging), at the end of chemoradiotherapy, every 6 weeks during the first year of consolidation, and every 12 weeks thereafter until the end of follow-up. Suspected pseudoprogression is adjudicated using iRECIST as a prespecified exploratory analysis, and progressions identified only on PET are confirmed by CT when feasible. All imaging for the primary endpoint will be reviewed by blinded independent central review (BICR); censoring rules, handling of missed or delayed assessments, and sensitivity analyses for informative censoring are prespecified in the statistical analysis plan. Safety will be monitored continuously, with adverse events graded by NCI CTCAE v5.0. Regular laboratory tests, cardiac markers, thyroid function, and pulmonary function tests will be performed. HRQoL questionnaires will be administered at key timepoints. Questionnaire compliance will be summarised by arm and time-point, with reasons for non-completion documented in eCRF. No imputation for primary descriptive analyses; multiple imputation under MAR and MNAR sensitivity analyses will be performed for longitudinal analyses, and subjects with >50% missing assessments will be flagged. Linear mixed-effects models will be used for repeated HRQoL outcomes, reporting least-squares mean changes with 95% CI. A ≥10-point change serves as the threshold for clinically meaningful change per EORTC references, with corresponding proportions summarised by arm. For biomarker research, tumor tissue (for genomics), serial blood samples (for ctDNA and immune profiling), and stool samples (for microbiome) will be collected at predefined intervals.

### Statistical analysis

All statistical analyses will be conducted using SAS 9.4 (or higher) or R software. The full statistical analysis plan is available from the corresponding author on reasonable request. The primary analysis will be based on the Intention-to-Treat (ITT) population.

#### Sample size

BEAM-LUNG is designed as a signal-seeking randomized phase II trial intended to inform a subsequent phase III trial, rather than as a formal superiority trial. For the control arm, a median PFS of 10 months is assumed based on the R-ALPS trial, in which benmelstobart monotherapy consolidation after chemoradiotherapy yielded a median PFS of 9.7 months (11). A clinically meaningful treatment effect of HR 0.55 for the experimental arm (median PFS approximately 18 months) is assumed. Using a two-sided log-rank test at α = 0.05 with 80% power, 88 PFS events are required (Schoenfeld method). With 152 patients (76 per arm), an accrual period of 30 months, a minimum follow-up of 12 months, and an anticipated PFS event proportion of approximately 70% (allowing for a 5% rate of loss to follow-up or non-evaluability), approximately 106 events are expected; the primary analysis is therefore triggered when 88 PFS events have been observed. No interim efficacy or futility analysis is planned; planned safety reviews after the first 20 and 40 patients are described under Monitoring.

- Efficacy Endpoints: Time-to-event endpoints (PFS, OS) will be estimated using the Kaplan-Meier method and compared using a stratified log-rank test. Hazard ratios (HR) and corresponding 95% confidence intervals (CI) will be estimated using a Cox proportional hazards model. The proportional-hazards assumption will be assessed by inspection of log(−log(survival)) plots and testing of time-dependent covariates. Patients who switch treatment, undergo off-protocol surgery, or never start chemoradiotherapy are analyzed as randomized within the ITT population; censoring-at-switch and per-protocol sensitivity analyses are prespecified. For local control endpoints, cumulative-incidence analyses treating death without progression as a competing event are prespecified as sensitivity analyses.
- Binary Endpoints: ORR, DCR, and other binary endpoints will be presented with 95% confidence intervals using the Clopper-Pearson method. Comparisons between arms will be performed using the Chi-square test or Fisher’s exact test as appropriate.
- Safety Analysis: Safety will be evaluated in the Safety Population (all patients who received at least one dose of study treatment). Adverse events will be summarized using descriptive statistics.
- Subgroup Analyses: Subgroup analyses are planned for exploratory purposes to assess the consistency of the treatment effect across key baseline characteristics: age, sex, histology, PD-L1 expression (TPS <1% vs ≥1% vs ≥50%), disease stage (IIIA vs IIIB–IIIC), N2 vs N3 disease, and radiotherapy and chemotherapy regimen. A prespecified test of the treatment-by-PD-L1 interaction will be performed; given the phase II sample size, all subgroup results are interpreted as hypothesis-generating. To limit multiplicity, secondary endpoints are tested in a prespecified order, and exploratory endpoints are explicitly labeled as such without adjustment.

#### Monitoring

Given the novelty of the induction triplet before thoracic chemoradiotherapy, an independent safety monitoring committee (ISMC), comprising two independent oncologists and one independent statistician, has been established. The ISMC reviews cumulative safety data after the first 20 and the first 40 enrolled patients have completed chemoradiotherapy, against predefined safety stopping boundaries (including rates of grade ≥4 pneumonitis, treatment-related death, fistula formation, and fatal hemorrhage). Trial conduct, including protocol compliance, data quality, and patient safety, will be monitored by the sponsor or designated contract research organization through regular site monitoring visits. Source data verification will be performed to ensure the accuracy of the data recorded in the electronic case report forms.

## Discussion

The BEAM-LUNG trial is designed to address a pivotal question in the post-PACIFIC era: can moving immunotherapy and targeted therapy earlier into the treatment sequence—specifically, into the induction phase—improve outcomes for patients with unresectable stage III NSCLC compared to the current standard of care?

The BEAM-LUNG trial incorporates several innovative elements that distinguish it from prior studies. First, the addition of anlotinib to induction therapy represents a novel “immuno-targeted-chemotherapy” strategy. Anlotinib, a multi-target tyrosine kinase inhibitor, not only exerts direct anti-tumor effects but also modulates the tumor immune microenvironment (14–16). By promoting tumor vascular normalization and converting immunologically “cold” tumors into “hot” tumors, anlotinib may overcome primary resistance to immunotherapy and enhance the efficacy of PD-L1 blockade. The combination of anlotinib with immune checkpoint inhibitors has demonstrated improved outcomes in NSCLC patients, and the R-ALPS trial has established benmelstobart with or without anlotinib as an effective consolidation option (11). Extending this combination into the induction phase represents a logical next step.

### Safety of anlotinib before thoracic chemoradiotherapy

The induction triplet has an established safety record: benmelstobart plus anlotinib and platinum-based chemotherapy has been evaluated in a phase 2 study in esophageal squamous cell carcinoma (19) and in a randomized phase 3 trial in extensive-stage small-cell lung cancer (12), without unexpected overlapping toxicity. Nevertheless, anti-angiogenic TKI exposure before thoracic radiotherapy raises specific concerns—bleeding, tumor cavitation, fistula formation, and vascular complications—and the vascular exclusion criteria necessarily render many patients with LA-NSCLC ineligible, which we acknowledge as a limitation. The protocol mitigates these risks through a minimum 7-day anlotinib washout before radiotherapy, mandatory post-induction radiological reassessment excluding new cavitation, fistula, or vessel encasement, baseline exclusion of patients with hemoptysis, vessel encasement, or significant cavitation, predefined management algorithms for hypertension, thrombosis, hemoptysis, pneumonitis, and esophageal toxicity, rules for delaying or omitting chemoradiotherapy, and independent safety monitoring committee review against predefined stopping boundaries (see Monitoring).

Anlotinib is deliberately restricted to the induction phase rather than continued during consolidation. Although R-ALPS supports benmelstobart–anlotinib consolidation (11), prolonging anti-angiogenic exposure beyond radiotherapy would increase cumulative toxicity (hypertension, proteinuria, thromboembolism, and impaired tissue healing) and would confound attribution of the induction effect. Restricting anlotinib to induction exploits vascular normalization and immune priming when they are biologically most useful, limits cumulative anti-angiogenic exposure, and keeps the consolidation phase identical in both arms.

Second, the trial design explicitly withholds benmelstobart during the radiotherapy phase. This approach is informed by the PACIFIC-2 and CheckMate 73L experiences (6, 7), which suggested that concurrent immunotherapy with cCRT may increase toxicity without improving efficacy. Most recently, the phase III ECOG-ACRIN EA5181 trial (N = 662) likewise showed that adding concurrent durvalumab to chemoradiotherapy did not improve overall survival (HR 1.03) or PFS (HR 1.05) compared with chemoradiotherapy followed by consolidation durvalumab (17), reinforcing the argument that concurrent checkpoint inhibition during cCRT is ineffective—and a fortiori that withholding benmelstobart during radiotherapy is justified. By temporally separating immunotherapy from radiotherapy, we aim to preserve the immune-priming benefits of induction therapy while minimizing the risk of radiation-induced pneumonitis and lymphopenia—toxicities that can compromise treatment delivery and patient outcomes.

Third, the randomized controlled design allows for direct comparison with the current standard of care. Unlike single-arm studies, this design will provide robust evidence regarding the incremental benefit of the induction strategy.

The choice of benmelstobart is supported by its established efficacy and safety profile (12, 13, 19). As a novel PD-L1 inhibitor, benmelstobart has demonstrated significant clinical activity in NSCLC, with the R-ALPS trial showing substantial PFS benefits as consolidation therapy. The inclusion of multiple, clinically guided radiotherapy dose-fractionation schedules enhances the trial’s practicality and generalizability to real-world decision-making.

The choice of benmelstobart rather than durvalumab as the common backbone reflects both the study context and the scientific question. Benmelstobart is approved in China in combination with anlotinib and chemotherapy for extensive-stage small-cell lung cancer, and R-ALPS has demonstrated its efficacy as consolidation after chemoradiotherapy in unresectable stage III NSCLC (11), with a PFS benefit of a magnitude comparable to that of durvalumab in PACIFIC. Thus, benmelstobart injection obtained a new indication approval from China’s National Medical Products Administration (NMPA); this approval applies to unresectable stage III NSCLC patients without known EGFR sensitizing mutations or ALK rearrangements who remain free of disease progression after platinum based concurrent or sequential chemoradiotherapy. Because both arms receive the same PD-L1 inhibitor, the trial provides an internally controlled estimate of the incremental value of the induction phase within a benmelstobart-based strategy; it does not compare the complete experimental strategy against the internationally established PACIFIC regimen, and the text has been revised to make this explicit. We acknowledge that a comparison against chemoradiotherapy plus durvalumab would be of international interest; this question is reserved for the subsequent phase III trial that BEAM-LUNG is designed to inform.

This trial has several limitations. As a phase II, signal-seeking study, it is not designed to provide definitive evidence of survival superiority, nor can it separately discern the contribution of induction chemoimmunotherapy from that of anlotinib; decomposing the induction strategy into its components would require a larger factorial phase III design, which this trial is intended to inform. The sample size, while providing 80% power for the primary endpoint, may limit the ability to detect differences in rare adverse events or in subgroup analyses. The open-label design introduces potential bias; this is mitigated by blinded independent central review of the primary endpoint. Radiotherapy heterogeneity has been reduced by the standardized prescription and prospective central quality assurance, but residual variation in chemotherapy choice must be considered when interpreting outcomes. Finally, the vascular exclusion criteria—necessary because of anlotinib—restrict generalizability to the broader population of patients with locally advanced NSCLC.

Despite these limitations, BEAM-LUNG provides a structured framework to evaluate a rational and innovative treatment paradigm. The comprehensive translational biomarker program—encompassing genomics, proteomics, single-cell sequencing, and gut microbiome analysis—is integral to this exploration, aiming to move beyond empirical combination towards identifying predictive biomarkers for patient selection. Coupled with rigorous quality-of-life assessments, the trial seeks to provide a holistic view of the treatment’s impact.

In conclusion, the BEAM-LUNG trial proposes a logical and refined strategy for unresectable stage III NSCLC. It directly confronts the key unanswered questions of timing and optimal combination by integrating induction immuno-targeted-chemotherapy for systemic and debulking effects with carefully sequenced radiotherapy designed to maximize efficacy while minimizing toxicity. The results may offer a new blueprint for synergizing these modalities, aiming to improve survival while upholding patient safety and treatment tolerability.
