# Supplementary material for: Efficacy and safety of induction benmelstobart plus anlotinib and chemotherapy followed by concurrent chemoradiotherapy and benmelstobart consolidation versus concurrent chemoradiotherapy followed by benmelstobart consolidation in patients with unresectable stage III NSCLC: a randomized, controlled, multicenter phase II trial protocol (BEAM-LUNG)
Source: Front Immunol. 2026 Sep 25;17:1915557. doi: 10.3389/fimmu.2026.1915557 (PMC13649746; doi:10.3389/fimmu.2026.1915557)
Supplement: Supplementary file 1 [file SupplementaryFile1.zip › Supplementary Figure S1.docx]

**筛选期和治疗期的研究流程**

| **研究时期** | **筛选期** | | **治疗期** | | | | | **研究治疗终止** **1** | **随访期** 2 | | |
| --- | --- | --- | --- | --- | --- | --- | --- | --- | --- | --- | --- |
|  |  |  | **诱导治疗** | | **同步放化疗** | | **维持治疗** |  |  |  |  |
| **治疗周期/访视名称** | 筛选期 | | 第 1 周期 | 第 2 周期 | 第 3 周期 | 第 4 周期 | 维持 n 周期 | 研究治疗终止 | 安全性随访末次 治疗后 30 天 | 安全性随访 末次治疗后 90 天 | 事件随访 3 （每 12 周） |
| **窗口期** **（天）^4^** | -28 至-8 | -7 至-1 | D1 | ± 3 | ± 3 | ± 3 | ± 3 | + 7 | ± 7 | ± 7 | ± 7 |
| **研究管理流程** | | | | | | | | | | |  |
| 知情同意书 | **X** | |  |  |  |  |  |  |  |  |  |
| 入排标准 | **X** | |  |  |  |  |  |  |  |  |  |
| 受试者身份卡发放 | **X** | |  |  |  |  |  |  |  |  |  |
| 人口统计学和病史 | **X** | |  |  |  |  |  |  |  |  |  |
| 既往治疗和合并治疗 5 | **X** | | **X** | **X** | **X** | **X** | **X** | **X** | **X** | **X** |  |
| 收集PDL1及EGFR 、 ALK 等驱动基因  检测结果 | **X** | |  |  |  |  |  |  |  |  |  |
| **临床操作/评估** | | | | | | | | | | |  |
| 入排标准核对 |  | **X** |  |  |  |  |  |  |  |  |  |
| AE/SAE 评估 6 | **X** | | **X** | **X** | **X** | **X** | **X** | **X** | **X^6^** | **X^6^** |  |
| 生活质量表&汉密顿抑郁量表^7^ | **X** | |  |  | **X** |  | **X^7^** | **X** |  |  |  |
| 超声心动图 | **X** | |  |  |  |  |  |  |  |  |  |
| 十二导联心电图 | **2X** | |  | **X** | **X** | **X** | **X** | **X** | **X** |  |  |
| 全套体格检查 | **X** | |  | **X** | **X** | **X** | **X** | **X** | **X** |  |  |
| 症状导向的体格检查 | **X** | |  | **X** | **X** | **X** | **X** | **X** | **X** |  |  |
| 身高，体重和生命体征 8 |  | **X** |  | **X** | **X** | **X** | **X** | **X** | **X** | **X** |  |
| ECOG 评分 |  | **X** |  | **X** | **X** | **X** | **X** | **X** | **X** | **X** |  |
| 后续抗肿瘤治疗 |  | |  |  |  |  |  |  | **X** | **X** | **X** |
| 生存状态 |  | |  |  |  |  |  |  |  |  | **X** |
| **抗肿瘤药物** **9** | | | | | | | | | | |  |
| 贝莫苏拜单抗 |  | | **X** | **X** |  |  | **X** |  |  |  |  |

| **研究时期** | **筛选期** | | **治疗期** | | | | | **研究治疗终止** **1** | **随访期** 2 | | |
| --- | --- | --- | --- | --- | --- | --- | --- | --- | --- | --- | --- |
|  |  |  | **诱导治疗** | | **同步放化疗** | | **维持治疗** |  |  |  |  |
| **治疗周期/访视名称** | 筛选期 | | 第 1 周期 | 第 2 周期 | 第 3 周期 | 第 4 周期 | 维持 n 周期 | 研究治疗终止 | 安全性随访末次 治疗后 30 天 | 安全性随访 末次治疗后 90 天 | 事件随访 3 （每 12 周） |
| **窗口期** **（天）^4^** | -28 至-8 | -7 至-1 | D1 | ± 3 | ± 3 | ± 3 | ± 3 | + 7 | ± 7 | ± 7 | ± 7 |
| 白蛋白紫杉醇/紫杉醇/紫杉醇脂质体+卡 铂/顺铂（非腺癌） |  | | **X** | **X** | **X** | **X** |  |  |  |  |  |
| 培美曲塞+卡铂/顺铂（腺癌） |  | | **X** | **X** | **X** | **X** |  |  |  |  |  |
| **放射治疗** |  | |  |  |  |  |  |  |  |  |  |
| 定位 CT |  | |  |  | **X** |  |  |  |  |  |  |
| 放射治疗 |  | |  |  | **X** | **X** |  |  |  |  |  |
| **实验室操作/评估：研究中心开展** | | | | | | | | | | |  |
| 妊娠试验 10 |  | **X** |  |  | **X** |  | **X10** | **X** | **X** |  |  |
| 血常规，血生化，凝血，尿常规 11 |  | **X** |  | **X** | **X** | **X** | **X** | **X** | **X** | **X** |  |
| 便常规（含潜血）^11^ |  | **X** |  | **X** | **X** | **X** | **X** | **X** |  |  |  |
| 垂体功能 12 | **X** | |  |  | **X** |  | **X12** | **X** | **X** | **X** |  |
| 心功能标志物 13 | **X** | |  | **X** | **X** | **X** | **X** | **X** |  |  |  |
| 胰酶（胰蛋白酶、血清淀粉酶、脂肪酶）  13 | **(X)** | | **(X)** | **(X)** | **(X)** | **(X)** | **(X)** | **(X)** |  |  |  |
| HBsAg/HBsAb/HBcAb，若HBsAg 或，HBcAb（+ ）需测 HBV DNA^14^ | **X** | |  |  |  |  |  | **X** |  |  |  |
| 若基线时：HBV DNA（－），且 1.HBsAg （+），或者 2.HBcAb（+）、HBsAg（－）， 则需在治疗期（每2周期）及治疗终止检测 HBV-DNA^14^ |  | |  |  | **X** |  | **X14** | **X** |  |  |  |
| HCV 抗体 14 | **X** | |  |  |  |  |  |  |  |  |  |
| HIV 抗体 | **X** | |  |  |  |  |  |  |  |  |  |
| 肿瘤标志物 15 | **X** | |  |  | **X** |  | **X15** | **X** |  |  |  |

| **研究时期** | **筛选期** | | **治疗期** | | | | | **研究治疗终止** **1** | **随访期** 2 | | |
| --- | --- | --- | --- | --- | --- | --- | --- | --- | --- | --- | --- |
|  |  |  | **诱导治疗** | | **同步放化疗** | | **维持治疗** |  |  |  |  |
| **治疗周期/访视名称** | 筛选期 | | 第 1 周期 | 第 2 周期 | 第 3 周期 | 第 4 周期 | 维持 n 周期 | 研究治疗终止 | 安全性随访末次 治疗后 30 天 | 安全性随访 末次治疗后 90 天 | 事件随访 3 （每 12 周） |
| **窗口期** **（天）^4^** | -28 至-8 | -7 至-1 | D1 | ± 3 | ± 3 | ± 3 | ± 3 | + 7 | ± 7 | ± 7 | ± 7 |
| 肺功能 16 | **X** | |  |  | **X** |  | **X16** | **X** | **X16** | **X16** |  |
| **疗效评价** | | | | | | | | | | |  |
| 影像学检查 17 | **X** | |  |  | **X** |  | **X17** | **X** | **(X)** |  | **(X)** |
| **生物标志物样本收集** | | | | | | | | | | |  |
| 肿瘤组织样本 18 | **X18** | |  |  |  |  |  |  |  |  |  |
| 血样 19 | **X** | |  |  | **X** |  | X19 |  |  |  |  |
| 粪便^20^ | **X** | |  |  | **X** |  | **X^20^** |  |  |  |  |

1. 受试者因任何原因终止研究治疗，应尽可能进行研究治疗终止访视；如果发生在末次研究治疗后 30 天 （ ±7 天），则无需进行末次治疗后 30 天的安全性随访。本研究允许重复筛选。

2. 因除疾病进展以外的原因停止用药的受试者，尽量按照既定的时间继续进行影像学评估，直至疾病进展（包括第一次 PD 、第二次PD（如果有））、开始新的抗肿瘤治疗、撤回知情同意、死亡或本研究

结束（以先发生者为准）。

3. 受试者开始新的抗肿瘤治疗或经研究者评价疾病进展后（以先发生者为准），应每 12 周±7 天通过电话随访受试者的生存情况；可酌情增加生存随访的频率；受试者完成最长至受试者首次接受试验用

药后 5 年的生存随访后即不再继续随访。

4. 筛选期的窗口期为 28 天，除首次给药外，治疗期的窗口期为±3 天（其中肿瘤评估窗口期为±7 天），研究治疗终止访视的窗口期为＋7 天，随访期的窗口期为±7 天。筛选期 ECOG 体力状况评分、

妊娠试验、血常规、血生化、凝血、尿常规、T3 或 FT3 、FT4 、TSH 需记录**给药前**数据并符合相应入排标准方可入组。

5. 记录从签署ICF 前 30 天的既往治疗，并记录签署ICF 后直至末次药物治疗后 90 天（或开始新的抗肿瘤治疗（以先发生者为准））的所有合并治疗；**之后需记录针对抗肿瘤药物相关的** **SAE 的合并治**

**疗**。

6. AE 从签署 ICF 开始记录直至末次药物治疗后 30 天（或开始新的抗肿瘤治疗（以先发生者为准）），SAE 从签署 ICF 开始记录直至末次药物治疗后 90 天（或开始新的抗肿瘤治疗（以先发生者为

准）。

7. 汉密顿抑郁量表和生活质量量表在筛选期，以及后续肿瘤疗效评估周期时进行。直至研究治疗终止。治疗终止访视时若过去 6 周内未进行过评估，则需要在此访视时进行一次生活质量评估。

8. **身高检查仅在筛选期进行**；生命体征包括体温、脉搏、呼吸频率和血压。在每次给药前测量体重，给药剂量按最新体重计算。

9. 药物在所有的临床和实验室操作/评估完成后，按试验方案给药 ，**每** **3 周**为一个给药周期。

10. 育龄期女性须进行血清或尿妊娠检验。其中，治疗期在**每** **2 个周期给药前（除外首次给药）3 天内**检测。分析将在当地研究中心进行。

11. 首次给药前的实验室检查应尽可能在给药前7天内进行检测，若实验室检查本中心报告时，可由研究者判断是否需要再次检查，不受7天内限制。常规实验室检查包括血常规、血生化、尿常规、凝血功 能。血常规检查项目包括全血细胞计数分类；血生化检查项目包括血液尿素/尿素氮，肌酐，钠，钾，氯，钙，血糖，总胆红素，直接胆红素，丙氨酸氨基转移酶，天冬氨酸氨基转移酶，碱性磷酸酶，乳酸脱 氢酶，总蛋白，白蛋白；尿常规检查项目包括比重，pH 值，尿糖， 尿蛋白，尿管型，酮体和血细胞；凝血功能包括活化部分凝血活酶时间（APTT）、凝血酶原时间（PT）、国际标准化比值（INR）。在 **每周期给药前** **3 天内（除外首次给药）**进行；

12. 垂体功能检查包括碘塞罗宁（T3 或 FT3）、甲状腺素（T4 或 FT4）、促甲状腺激素（TSH）、生长激素（HGH）、皮质醇（CORT）、促肾上腺皮质激素（ACTH）分析。其中，若首次给药前已

有本中心报告时，可由研究者判断是否需要再次检查，治疗期在**每** **2 个周期给药前** **3 天（除外首次给药）内**检测。

13. 心功能标准物包括：心肌酶谱[肌酸激酶（CK）及其同工酶] 、肌钙蛋白（TnT 或 TnI）。若首次给药前已有本中心报告时，可由研究者判断是否需要再次检查，治疗期在**每周期给药前** **3 天（除外首**

**次给药）内**检测。分析将在当地研究中心进行。胰酶（胰蛋白酶、血清淀粉酶、脂肪酶）的检测为非必需检测项目，可根据当地研究中心的常规操作选择性检测。

14. 所有受试者在筛选期均需进行乙肝表面抗原（HBsAg）、乙肝表面抗体（HBsAb）和乙肝核心抗体（HbcAb）和丙型肝炎病毒（HCV）抗体检测，HBsAg（+ ）或 HBcAb（+ ），受试者须进一步进行乙 型肝炎病毒（HBV）DNA 滴度检测。若筛选期（基线）时：HBV DNA（－），且 1.HBsAg（+ ），或者 2.HBcAb（+ ），HBsAg（－），则需在治疗期**每** **2 个周期、治疗终止**检测 HBV DNA 。HCV抗体 阳性，需加做HCV-RNA。

15. 肿瘤标志物：癌胚抗原 （CEA）、 细胞角蛋白 19 片段 （CYFRA21-1）、 鳞状细胞癌相关抗原测定（SCC）、 神经元特异性烯醇化酶 （NSE）、 胃泌素释放肽前体 （Pro-GRP）。在筛选期、以 及后续肿瘤疗效评估周期时进行。

16. 肺功能检查：第 1 周期给药前、放疗前、放疗结束后 1个月内、放疗结束后 3个月 （ ±7 天）。

17. 肿瘤影像学评估：评估周期：筛选期、第三周期给药前、放疗结束后首次用药前、免疫维持阶段每6周±7天、免疫维持治疗结束后第一年内每3个月复查一次，一年后每半年复查一次，至多复查至免疫

维持治疗结束后5年。筛选期需进行以下部位：头部、颈部、胸部、腹部、盆腔（增强 CT 或 MRI），骨扫描（入组前 3 个月未进行全身骨扫描的患者，应行全身骨扫描，治疗期建议每 16 周期检查一 次，两次间隔不得超过 18 周期）。做过PET-CT后可不进行骨扫描及除可评估病灶部位以外的CT/MRI。外院做的PET-CT/骨扫描可由研究者判断是否需要在本中心再检查。在整个研究期间，同一部位的 检查方法应尽量保持一致；腹部扫描 CT 层厚 具体参见影像手册，动静脉期双相，空腹并饮水或造影剂 1000ml。

以上，建议冠状位及矢状位重建；如果无禁忌证，应使用造影剂。研究者根据 RECIST v1. 1 对肿瘤影像学情况进行评估（肿瘤评估可由研究者根据临床需要增加评估次数），研究者应根据自己的疗效评 价结果进行后续治疗的判断。如果在首次给药前 28 天内已进行肿瘤评价，且在本中心（PET-CT/骨扫描可不要求在本中心检查）使用同样的方法和机器，可作为基线肿瘤评价。终止访视时，如果前 4 周内已经进行过肿瘤影像学检查， 则终止访视可以不用再次进行肿瘤影像学检查。**因除疾病进展以外的原因停止用药的受试者，按照既定的时间继续进行影像学评估，**直至疾病进展、开始新的抗肿 瘤治疗、撤回 ICF、死亡或本研究结束（以先发生者为准）

**18.** 受试者**必须提供**诊断为NSCLC 时或之后（建议首次研究用药前 6 个月内），采集于病灶部位，经甲醛溶液固定－石蜡包埋（FFPE）处理的肿瘤样本（石蜡块或未染色切片）及上述标本的相关病理报 告。如果受试者没有存档的符合要求的肿瘤组织样本，则必须接受筛选期进行的新鲜肿瘤病灶活组织检查，以获得相应肿瘤样本（获取标本的数量根据活检情况决定）。肿瘤组织切片样本将用于中心试验 室的MRD 、PD-L1 的表达水平、MSI 、TMB 检测。新鲜采集标本、切除术、空心针芯活检、切除、切开、冲压或钳夹活检均在可接受范围内。不接受针吸样本（即缺乏完整的组织结构仅提供细胞悬 液和/或细胞涂片的样本）、刷涂样本、来自胸腔或腹水的细胞沉淀样本。**组织样本要求详见实验室操作手册。**

19. 血样采集：第 1 周期给药前、放疗前、放疗结束后 3 个月 （ ±7 天）、放疗结束后 1 年 （±7 天）。将用于 MRD 、PD-L1 的表达水平、MSI 、TMB 检测。重复筛选的受试者无需再次进行 PD-L1 检测。

20. 粪便采集：第 1 周期给药前、放疗前、放疗结束后 3 个月 （ ±7 天）、放疗结束后 1 年 （±7 天）。将用于肠道微生物检测。
